# Response of Glacier and Lake Dynamics in Four Inland Basins to Climate Change at the Transition Zone between the Karakorum And Himalayas

**DOI:** 10.1371/journal.pone.0144696

**Published:** 2015-12-23

**Authors:** Zhiguo Li, Kuangsheng Fan, Lide Tian, Benlin Shi, Shuhong Zhang, Jingjing Zhang

**Affiliations:** 1 College of Environment and Planning, Shangqiu Normal University, Shangqiu, China; 2 Institute of Tibetan Plateau Research, Chinese Academy of Sciences, Beijing, China; 3 College of Life Science, Shangqiu Normal University, Shangqiu, China; Institute of Tibetan Plateau Research, CHINA

## Abstract

Inland glacier and lake dynamics on the Tibetan Plateau (TP) and its surroundings over recent decades are good indicators of climate change and have a significant impact on the local water supply and ecosystem. The glacier and lake changes in Karakoram are quite different from those of the Himalayas. The mechanisms of the complex and regionally heterogeneous behavior of the glacier and lake changes between the Karakorum and Himalayas are poorly understood. Based on satellite images and meteorological data of Shiquanhe, Hetian, and Yutian stations, we demonstrate that the overall retreat of glaciers and increase of lake area at the transition zone between the Karakoram and Himalayas (TKH) have occurred since 1968 in response to a significant global climate change. Glacial areas in the Songmuxi Co basin, Zepu Co basin, Mang Co basin and Unnamed Co decreased by -1.98 ± 0.02 km^2^, -5.39 ± 0.02 km^2^, -0.01 ± 0.02 km^2^, and -0.12 ± 0.02 km^2^ during the study period, corresponding to losses of -1.42%, -2.86%, -1.54%, and -1.57%, respectively. The lake area of the Songmuxi Co, Zepu Co, Mang Co and Unnamed Co increased by 7.57 ± 0.02 km^2^, 8.53 ± 0.02 km^2^, 1.35 ± 0.02 km^2^, and 0.53±0.02 km^2^, corresponding to growths of 30.22%, 7.55%, 11.39%, and 8.05%, respectively. Increases in temperature was the main reason for glacier retreat, whereas decreases in potential evapotranspiration of lakes, increases in precipitation, and increases in melt water from glaciers and frozen soil all contributed to lake area expansion.

## Introduction

The TP and its surroundings contain the largest number of glaciers outside of polar regions and is often called the world’s "Third Pole" [1]. Glacier and lake changes on the TP can change atmospheric circulation patterns and affect agriculture, power generation, and the water supplies of 1.5 billion people across ten countries [2–4]. Expanding inland lake areas have a substantial impact on local ecosystems and pastures [5]. The changes of the TP glaciers and lakes exhibit regionally heterogeneous behavior [1, 6]. The glaciers in the eastern, central and western Himalayas in the southern part of the TP all showed retreating trends [7–9], whereas the Karakoram glaciers in the northern part of the TP are considered to be in a stable or advancing state [1, 10, 11]. The glacial lakes in the eastern, central and western Himalayas in the southern part of the TP all showed expansion trends [8, 9, 12–16], whereas the larger lakes showed shrinking trends [9, 13, 17–20]; meanwhile, the Karakoram glacial lakes in the northern part of the TP are reported to shrinking trend [14] while larger lakes are considered to be in a stable state [21]. The Songmuxi Co, Zepu Co, Mang Co and Unnamed Co are located in TKH and are covered by different glacier percentages. In the four basins, it is unclear whether glaciers advanced, retreated, or remained unchanged or whether the corresponding lakes shrank, expanded, or remained stable. Thus, the status of glaciers and lakes, as well as the relationship between glacier and lake changes remain unclear because of insufficient research. Considering the scarcity of systematic observations of the study area, remote sensing images based on multiple datasets and GIS technology were applied to reveal the process of glacier and lake changes in TKH, and the interactions between glaciers and lakes. The results of this study may help further the understanding of the spatial pattern and mechanism of glacier and lake changes in the TP and can provide a reference for developing response measures against issues related to these changes and preserving the living conditions of local residents.

## Materials and Methods

### Study area

**Fig 1:**
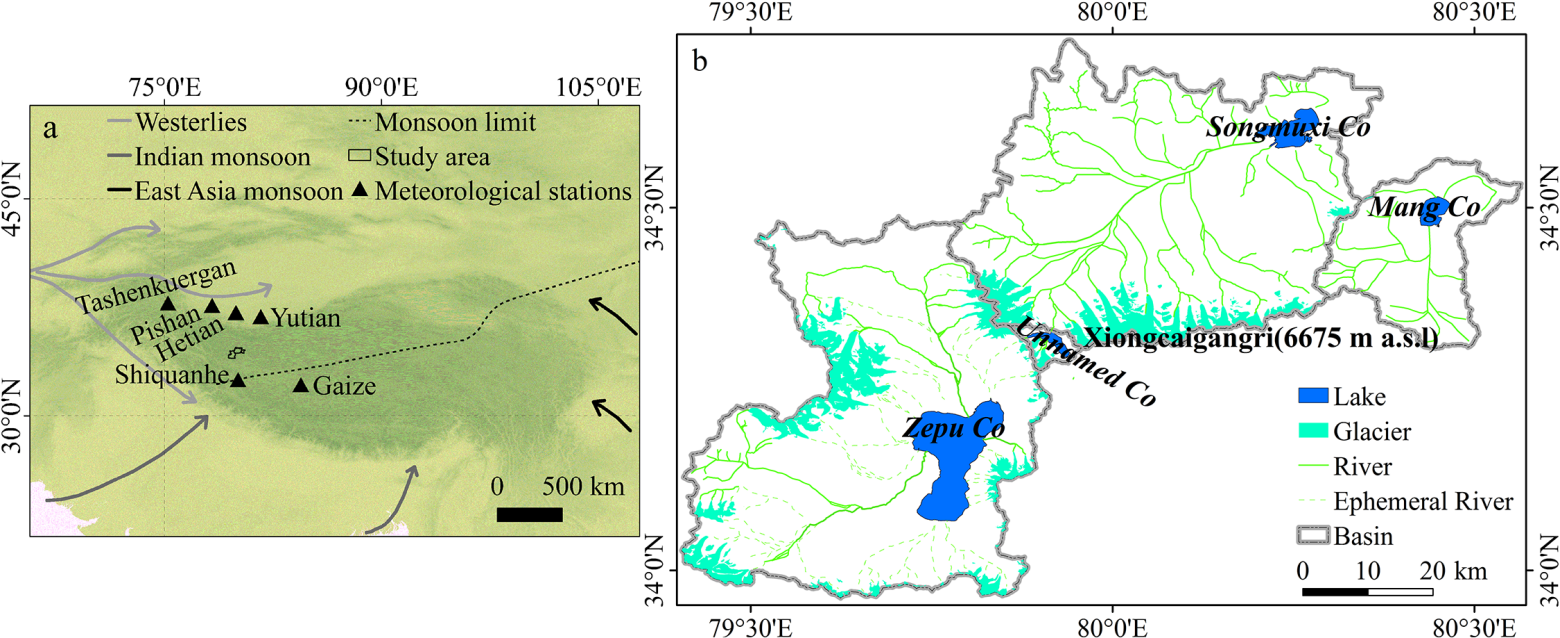
Location of the study area: (a) The location of the study area within the TP and the meteorological stations. Dashed line shows the average monsoon margin, which may fluctuate with the seasons and year. (b) Distribution of glaciers and lakes in the four basins.

The Songmuxi Co, Zepu Co, Mang Co and Unnamed Co lie in the south of Karakorum, north of the western Himalayas, and are situated on the northwestern part of the Tibetan Plateau (Fig 1). The Asian monsoons and the westerlies influence the climate in these four basins. The climate is a transitional climate ranging from a plateau arid sub-frigid temperate climate and a plateau arid climatic transition zone [22], and the local glaciers have been classified as sub continental-type glaciers [1, 23]. The glaciers and lakes provide a fresh water source for the local environment as well as the farmers and herdsmen. The mean equilibrium line attitude (ELA) of the glaciers in the four basins lies at an elevation of approximately 5900 m [1, 23].

There is no meteorological station in the four basins. Temperature and precipitation data from the nearest stations (Shiquanhe station, Hetian station and Yutian station) were used to analyze the local climate. The long-term annual average temperature at Shiquanhe station, Hetian station and Yutian station was 0.8°C, 12.9°C and 11.8°C, respectively; the long-term annual precipitation at Shiquanhe station, Hetian station and Yutian station was 71.6 mm, 40.3 mm and 51.4 mm, respectively. The rainfall at Shiquanhe station, Hetian station and Yutian station was concentrated in May to September, accounting for 88.6%, 71.5% and 80.7%, respectively.

### Remote sensing data

This study utilized a total of 20 topographic maps which were acquired in 1968 and derived from aerial photographs taken by the State Bureau of Surveying and Mapping. Our study scanned the maps and implemented the results to the Universal Transverse Mercator (UTM) coordinate system and the World Geodetic System 1984 ellipsoidal elevation (WGS84) using kilo–grids and ENVI/IDL 5.1 software (with root–mean–square errors (RMSE) less than 2 m in both the x and y directions). The rectified topographic maps were cut and spliced (hereinafter referred to as RTMS). The RTMS were used to extract glacier and lake cover. Moreover, the RTMS were also exploited to generate a digital elevation model (DEM) (hereafter referred to as 1968DEM based on 10-m interval contours and digitized spot heights). The datum transformation method provided errors of <0.002 m [24]. The ridgeline vectors were yielded from 1968DEM and used to segment glaciers.

This study employed Landsat MSS/TM/ETM+/OLI images to extract glaciers and extent values and to monitor glacier changes (shown in Table 1). The US Geological Survey (USGS; http://glovis.usgs.gov) and the Global Land Cover Facility provided the Landsat images. The cloud cover of the images used to extract the glacier and lake extents in Table 1 is between 0% and 39%. The cloud masking had little impact on the delineation of glacial and lake outlines as little cloud coverage occurred over the glaciers and lakes in these images. All of the selected remote sensing images of area extraction were from around the end of the melt season, and two or three other images taken at nearly the same time provided reference data to help determine the seasonal snow cover and cloud coverage. Our study used manual digitization to delineate glacier boundaries, as recommended by [25] (this provided the best tool for extracting the most reliable information from satellite images).

**Table 1:** Data used for this study.

Data source	Acquisition Date	Path, Row	Sensor Resolution (m)	Utilization	Cloud Coverage(%)
Topographic map	1968		5	a, d	
Landsat MSS	01 Nov 1976	156/036	57	a, d	8
Landsat MSS	14 Oct 1976	156/036	57	d	10
Landsat MSS	07 Dec 1976	156/036	57	d	4
Landsat MSS	02 Nov 1976	157/036	57	a, d	4
Landsat MSS	20 Nov 1976	157/036	57	d	1
Landsat TM	19 Aug 1993	145/036	28.5	b, d	3
Landsat TM	17 Oct 1991	145/036	28.5	a, d	1
Landsat TM	10 Oct 1992	146/036	28.5	a, d	1
Landsat TM	29 Dec 1992	146/036	28.5	d	71
Landsat TM	25 Jul 1993	146/036	28.5	d	39
Landsat ETM+	08 Oct 2000	146/036	28.5	a, d	0
Landsat ETM+	25 Sep 2001	146/036	28.5	d	3
Landsat ETM+	06 Feb 2000	145/036	28.5	d	11
Landsat ETM+	20 Oct 2001	145/036	28.5	a, d	0
Landsat 8 OLI	01 Aug 2013	145/036	30	b, d	1
Landsat 8 OLI	27 Sep 2013	145/036	30	c, d	1
Landsat 8 OLI	05 Nov 2013	146/036	30	c, d	13

a indicates glacier and lake extraction

b indicates glacier extraction

c indicates lake extraction

and d indicates reference data.

### Meteorological data

No weather stations were operated within the four basins during our study period: the nearest three stations were Shiquanhe station (4278 m asl), Hetian station (1375 m asl) and Yutian station (1422 m asl). The meteorological data were downloaded from the China Meteorological Data Sharing Service System (http://cdc.nmic.cn/home.do). The meteorological data of the three stations were used to analyze climate changes and discern the causes for changes in the state of the glaciers and lakes from 1968 to 2013.

### Precision evaluation

The errors extracted from multi-temporal satellite images mainly resulted from (1) sensor resolution, (2) co-registration errors, and (3) boundary delineations. The first two types of errors were evaluated using a remote sensing uncertainty evaluation formula [9]. The resulting area uncertainty is 0.007, 0.013, 0.016, and 0.02 km^2^ between each pair of datasets (i.e. 1968–1976, 1976–1991, 1991–2001, 2001–2013), respectively.

The glacial delineation errors mainly resulted from the experience of the operator that delineated the glacier boundary, such as classifying the shadowed areas as perennial or seasonal snow. The changes in glacial area between the two adjacent images were delineated by two other independent colleagues to estimate the glacial area change delineation error: the differences between the different operators fell within 3%. Based on tests and analyses, the differences determined in glacial area caused by image quality (which can be affected by seasonal snow and shadow) fell below 2%.

### Glacier and lake area change rate calculation

Glacier and lake area change is the area difference between the two different periods. In this paper, changes in absolute area change rate and relative rates are calculated as follows.

$$V_{a}=\frac{A_{i}-A_{j}}{Y_{j-i}}$$ (1)

Vr=[(AjAi)1Yj−i−1]×100% (2)

Where *V*_*a*_ is absolute area change rate (km^2^/a) and *V*_*r*_ is relative area change rate (%/a), *A*_*i*_ and *A*_*j*_ is for the beginning area and end area, *Y*_*j-i*_ is the time interval of the area used of the data source gathered.

## Results

### Glacier area change in the four basins from 1968 to 2013

Table 2 summarizes the glacier changes in areal extent in the four basins. The total glacier area of the four basins decreased from 335.93 km^2^ in 1968 to 328.43±0.08 km^2^ in 2013, corresponding to a loss of -7.5±0.224 km^2^ of ice and an average loss rate of -0.17 km^2^ per year. The overall area loss percentage between 1968 and 2013 was -2.23%, and the average observed decrease of the glacier area was -0.05% per year. The glacier area of the Songmuxi Co basin, Zepu Co basin, Mang Co basin and Unnamed Co basin was 139.25 km^2^, 188.40 km^2^, 0.65 km^2^ and 7.63 km^2^ in 1968, respectively, with a loss of -1.98±0.02 km^2^, -5.39±0.02 km^2^, -0.01±0.02 km^2^ and -0.12±0.02 km^2^, respectively, corresponding to a loss of -1.42%, -2.86%, -1.54% and -1.57%, respectively. Table 3 shows the glacier area change percentage mean rate and parameters in the four basins from 1968 to 2013, and the glaciers in the four basins all show slight retreats. The lowest area change percentage mean rate of the Songmuxi Co basin may be for the largest average area. Although the glaciers in the Mang Co basin had the smallest average glacier area, these glaciers had the highest average elevation of the glaciers in the four basins and were difficult to melt due to the low temperature (calculated from elevation plus temperature lapse rate), so their area loss percentage was the second lowest. The reason why the glacier area loss percentage in the Zepu Co basin is comparatively large is because there are some small glaciers in the south of the basin, and these small glaciers influence more by the declining Asian monsoons [1] than the other glaciers in the north of the four basins, which led to more melt.

**Table 2:** Glacier area variation in the four basins from 1968 to 2013.

Year	Glacier area (km^2^)						
	Songmuxi Co	Zepu Co	Mang Co	Unnamed Co	Total	Change	Mean Change Rate (km^2^/a)
1968	139.25	188.40	0.65	7.63	335.93	/	
1976	139.22±0.007	187.93±0.007	0.65±0.007	7.63±0.007	335.43±0.028	-0.5±0.028	-0.063
1991	138.71±0.013	187.41±0.013	0.65±0.013	7.63±0.013	334.4±0.052	-1.03±0.052	-0.069
2001	138.38±0.016	186.19±0.016	0.64±0.016	7.62±0.016	332.83±0.064	-1.57±0.064	-0.157
2013	137.27±0.02	183.01±0.02	0.64±0.02	7.51±0.02	328.43±0.08	-4.4±0.08	-0.367
Total					/	-7.5±0.224	-0.167

**Table 3:** Glacier area change percentage mean rate and parameters in the four basins from 1968 to 2013.

Basin	Area change Percentage Mean rate (%/a)					Average glacier	
	1968–1976	1976–1991	1991–2001	2001–2013	1968–2013	area (km^2^)	elevation (m asl)
Songmuxi Co	-0.003	-0.025	-0.024	-0.067	-0.032	1.31	6005
Zepu Co	-0.031	-0.019	-0.065	-0.144	-0.065	1.00	6065
Mang Co	-0.000	-0.000	-0.155	-0.000	-0.034	0.11	6071
Unnamed Co	-0.000	-0.000	-0.013	-0.121	-0.035	0.69	5997

### Area change of Songmuxi Co, Zepu Co, Mang Co and Unnamed Co from 1968 to 2013

Table 4 summarizes the lake changes in areal extent of Songmuxi Co, Zepu Co, Mang Co and Unnamed Co from 1968 to 2013. The total lake area of the four increased from 156.45 km^2^ in 1968 to 174.44±0.08 km^2^ in 2013, corresponding to an increase of 17.99±0.224 km^2^ and an average expansion rate of 0.400 km^2^ per year. The overall area increase percentage between 1968 and 2013 was 11.50%, and the average observed increase in lake area was 0.05% per year. The lake area of Songmuxi Co, Zepu Co, Mang Co and Unnamed Co was 25.05 km^2^, 112.98 km^2^, 6.58 km^2^ and 11.85 km^2^ in 1968, respectively, with increases of 7.57±0.02 km^2^, 8.53±0.02 km^2^, 1.35±0.02 km^2^ and 0.53±0.02 km^2^, respectively, corresponding to growth of 30.22%, 7.55%, 11.39% and 8.05%, respectively. Table 5 shows the lake area variation, with all four lakes showing expansion trends (S1 Dataset). The highest area change percentage mean rate of Songmuxi Co basin may because of the larger basin area, larger glacier area change or the lower basin elevation.

**Table 4:** Lake area variation of Songmuxi Co, Zepu Co, Mang Co and Unnamed Co from 1968 to 2013.

Year	Lake area (km^2^)						
	Songmuxi Co	Zepu Co	Mang Co	Unnamed Co	Total	Change	Mean Change Rate (km^2^/a)
1968	25.05	112.98	11.85	6.58	156.45	/	
1976	25.62±0.007	114.93±0.007	12.10±0.007	6.61±0.007	159.27±0.028	+2.82±0.028	+0.353
1991	25.86±0.013	114.64±0.013	12.24±0.013	6.88±0.013	159.62±0.052	+0.35±0.052	+0.023
2001	28.74±0.016	117.52±0.016	12.69±0.016	6.96±0.016	165.91±0.064	+6.29±0.064	+0.629
2013	32.62±0.02	121.51±0.02	13.20±0.02	7.11±0.02	174.44±0.08	+8.53±0.08	+0.711
Total					/	+17.99±0.224	+0.400

**Table 5:** Lake area change percentage mean rate and parameters of the four lakes from 1968 to 2013.

Basin	Area change(km^2^)	Area change Percentage Mean rate (%/a)					Basin	
		1968–1976	1976–1991	1991–2001	2001–2013	1968–2013	area (km^2^)	mean elevation (m asl)
Songmuxi Co	-1.98±0.02	+0.282	+0.062	+1.062	+1.061	+0.589	1698.92	5413
Zepu Co	-5.39±0.02	+0.214	-0.017	+0.248	+0.279	+0.162	1792.51	5480
Mang Co	-0.01±0.02	+0.261	+0.077	+0.362	+0.329	+0.240	403.95	5313
Unnamed Co	-0.12±0.02	+0.057	+0.267	+0.116	+0.178	+0.172	32.25	5830

## Discussion

### Climate change and impact on glacier and lake change

Due to the lack of continuous detailed observation site data of weather, glaciers, permafrost and hydrology in the study area, meteorological data from the nearest stations (Shiquanhe station, Hetian station and Yutian station) were used to analyze the local climate changes and their impact on glacier and lake changes.

**Fig 2:**
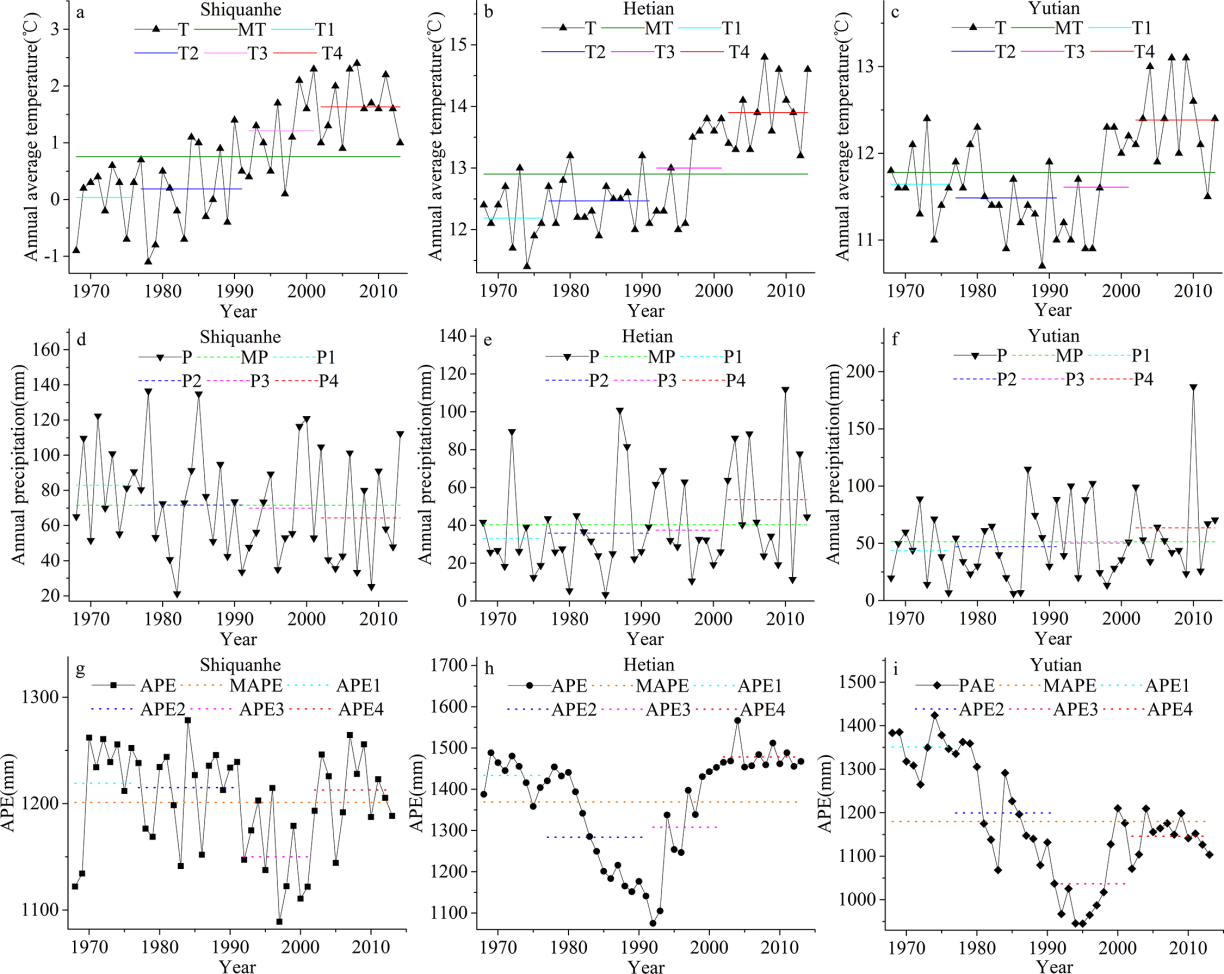
Variations in the MAT, AP and APE at the three stations from 1968 to 2013. a, b, c was MAT variations. d, e, f was AP variations. g, h, i was APE variations.

The mean annual temperature (MAT), annual precipitation (AP) and annual potential evapotranspiration (APE) data from the preceding three meteorological stations were used to assess the degree of climate change (Fig 2). In Fig 2, MMAT, MAP and MAPE refer to the MAT, AP and APE long term mean values (1968–2013), respectively; T1, T2, T3 and T4 denote the mean MAT value of 1968–1976, 1976–1991, 1991–2001 and 2001–2013, respectively, with similar labels for AP and APE.

From 1968 to 2013, the MAT of Shiquanhe station, Hetian station and Yutian station all showed an increasing trend; the rates were 0.052°C a^-1^ (R^2^ = 0.5522), 0.048°C a^-1^ (R^2^ = 0.5809) and 0.054°C a^-1^ (R^2^ = 0.1831), respectively (Fig 2A, 2B and 2C). The MAT of Shiquanhe station and Hetian station have continued increasing for the four phases, whereas the MAT of Yutian station declined from 1968 to 1976. Warming may lead to the melting of glaciers and permafrost, and increased melt water supply to lakes. The total MAT of the three stations showed increasing trends, with slopes of 0.119°C •a^-1^ (R^2^ = 0.565). Increases in temperature will increase glacier melt and mass loss.

From 1968 to 2013, the AP of Shiquanhe station showed a decreasing trend at a rate of -0.423 mm a^-1^ (R^2^ = 0.0352), whereas the AP of Hetian station and Yutian station showed increasing trends at rates of 0.476 mm a^-1^ (R^2^ = 0.0594) and 0.547 mm a^-1^ (R^2^ = 0.0453), respectively (Fig 2D, 2E and 2F). Comparing Fig 2 and Table 3, it can be found that the lake area expansion trend was consistent with the increasing AP of Hetian and Yutian station, which may represent the main watershed controlled by westerly circulation. The reason why the precipitation change of Shiquanhe station does not link with the lake area expansion trend may be because Shiquanhe station is located in the south of the four basins and more influenced by the south Asian monsoon. The total of P of the three stations showed increasing trends, with slopes of 0.600 mm•a^-1^ (R^2^ = 0.0183). The slightly increased precipitation may provide more mass supplies to glaciers, therefore, the glaciers of the four basins appeared slightly retreat.

Because the four lakes were inland lakes, their water loss depends mainly on lake evapotranspiration. The Penman-Monteith equation applied by Yao et al. [26] was employed to calculate the APE of the three stations; the results are shown in Fig 2G–2I. From 1968 to 2013, only the APE of Hetian station showed an upward trend, with a slope of 1.461 mm a^-1^ (R^2^ = 0.0242); the APE of Shiquanhe and Yutian showed decreasing trends, with slopes of -0.521 mm a^-1^ (R^2^ = 0.0206) and -6.341mm a^-1^ (R^2^ = 0.0453), respectively. The total of APE of the three stations showed decreasing trends, with slopes of -5.094 mm•a^-1^ (R^2^ = 0.086).

## Contribution of glaciers and permafrost to lake change

The annual average temperature of Shiquanhe station, Hetian station and Yutian station all showed increasing trends, which indicates that the melting of glaciers and permafrost was enhanced. A 1°C increase in temperature requires an approximate 25% [27] increase in precipitation to replenish a glacier’s corresponding mass loss. The 25% value was obtained through mass-balance modeling and it may be affected by differences in absolute precipitation. Under the combined effects of temperature and precipitation, the glaciers retreated continuously, although the rate varied during different phases. In addition, increasing temperatures can also reduce snow, thereby enhancing snowmelt by reducing the albedo and further promoting melting. Comparing Table 2 and Table 4, it can be seen that the lake area expansion corresponds to the glacier shrinkage during the four stages. However, Table 2 shows that the glaciers in the four basins only slightly retreated, and in the case of Mang Co, which has fewer glaciers, the lake area expanded rapidly, implying that the contribution of glacier melt to the lake area expansion was small. The conclusions are consistent with the findings of other researchers’ [18, 28]. The increased glacier contribution can be deduced by the data in Table 2 and the empirical formula used by Liu et al. [29], with an ice density of 0.9 g • cm^-3^; the results are shown in Table 6. The four basins are located in the middle portion of the continuous permafrost zone of western TP [28]. Although permafrost observations have not been made in the study area, the local temperature of the four basins could be assumed to be relatively low for high elevations (Table 5). Because the Shiquanhe station is the nearest meteorological station to the study area and its elevation is the highest, this station was selected as the reference and temperature-elevation gradients of 0.72°C (100 m)^-1^ (mean value of summer and autumn) [30] were selected to calculate changes in the number of positive days in the four basins. From 1968 to 2013, positive days in the Songmuxi Co, Zepu Co, Mang Co, and Unnamed Co basins increased by 17 d, 12 d, 20 d, and 28 d respectively. Air temperature was low and the total number of positive days was less in higher basins, preventing permafrost degradation even under high increases of positive days. In continuous permafrost in drought-arid areas, increased positive days may cause permafrost degradation; however, water released from the permafrost accounted for only a small proportion of groundwater flow. Thus, increases in permafrost meltwater must have been limited in the past. However, a continuous rise in temperature will induce further continuous permafrost degradation, and the contribution of shrink permafrost to lake water volume cannot be ignored in the long term.

**Table 6:** Increased glacier melt water of the four basins from 1968 to 2013.

Basin	Increased water equivalent of different period (×km^3^)				
	1968–1976	1976–1991	1991–2001	2001–2013	1968–2013
Songmuxi Co	+0.0110	+0.1862	+0.1204	+0.4039	+0.7215
Zepu Co	+0.1955	+0.2161	+0.5060	+1.3121	+2.2296
Mang Co	+0.0000	+0.0000	+0.0004	+0.0000	+0.0004
Unnamed Co	+0.0000	+0.0000	+0.0010	+0.0115	+0.0125

### Reasons for glacier and lake change in the TP

The current glacier and lake changes in the TP have large spatial heterogeneity. With regard to the glaciers, most of the glaciers of the Himalayas in the south of the TP retreat rapidly, whereas the glaciers of the Karakorum in the northern part of the TP advance or tend to be relatively stable. In terms of lake changes, the glacial lakes fed by a large proportion of glacier melt water expanded and the larger lakes supplied by a small proportion of glacier melt water diminished in the southern Himalayas [6], but some lakes in northern Karakorum shrunk [14] or remained stable [21]. This study shows that the glaciers in the four basins of the southern Karakoram showed a slight retreat and that the four lakes were significantly expanded. The slight shrink rate consistent with the findings of glacier elevation changes from the north bank of the Bangong Co Basin (roughly consistent with the study area of this research) [31] and West Kunlun Shan [32]. Climate change is an important factor for glacier and lake level changes [1, 2, 27]. Rising temperature and decreasing precipitation are the main reasons for glacial retreat, glacial lake expansion and large lake shrinkage [17, 18, 33, 34]. Other factors, such as dust and black carbon [35], affect the Himalayas glaciers due to the proximity to emission sources, thereby affecting the water cycle. Climate modeling studies show that the peculiar Karakoram glacier changes may be related to increased snowfall [34, 36], which changes the albedo and reduces solar radiation, thus reducing glacier shrinkage. Satellite-derived data also show the presence of cooling in high altitude Karakorum [33], which will also reduce ablation. The glaciers in the south of the four basins are close to Shiquanhe station, and rising temperatures and decreasing precipitation can be considered to be the causes of glacial retreat. From the point of view of atmospheric circulation, on the one hand, the four basins located in the interaction region of southwest monsoon and westerly circulation and the Asian monsoon recession [1] may lead to the retreat of glaciers; additionally, the southwest monsoon in South Asia may carry black carbon to the area and promote the melting of glaciers; on the other hand, because the studied area is located at the transition zone between the Karakorum and Himalayas and at the high elevation, the slightly increased snowfall may provide more mass supplies to glaciers, therefore, the glaciers of the four basins appeared slightly retreat. Increased precipitation led to most TP lake expansion [17–19], and the lake area increases of the four lakes are consistent with this trend. Currently, scholars have different conclusions on the reasons for the lake area change in the TP. Glacier meltwater may play a dominant role in the areal expansion of most glacial lakes in the TP [14–16]. For large lakes, the area changes have been driven mainly by changes in precipitation and evapotranspiration and not solely by the glacier meltwater. Yao et al. [26] suggested that the expansion of the Hoh Xil area lakes in the recent 40a was mainly due to increased precipitation and evaporation reduction and glacier meltwater increases and that the release of frozen soil moisture from climate warming is a secondary reason. Lei et al. [17], Song et al. [18] and Zhang et al. [19] proposed that although the loss of glaciers in the Tibetan plateau and decline of lake area evaporation contribute to the lake area growth, the overall lake area increase is mainly due to the significant increase in precipitation on the TP. Li et al. [20] suggested that the contribution of precipitation, evaporation, glacial melting and other factors change in different periods. Water balance level based on estimations of lake input (precipitation and melting) and output (evaporation) has not been discussed in this study because of limited data. Firstly, there are no long-term water level, permafrost, and runoff monitoring data. Secondly, the Hetian and Yutian stations are located at the northern slope or mountain foot of the western and middle Kunlun range while the studied area is located at the southern part of the mountain ranges. Moreover, these stations are at elevations of less than 2000 m asl, which is not suitable for water balance calculations for the study area at a high elevation. The climate trend of the study area could be reflected in the stations that are dominated similarly by westerlies. We observed climate trends of the Tashenkuergan, Pishan, Hetian, and Yutian stations (Fig 1A) and found that precipitation in these four stations all showed an increasing trend, which may reflect strengthened westerlies. In contrast, precipitation at the Shiquanhe station, which is located at the northern limit of the summer monsoon, showed a decreasing trend, possibly reflecting weakening Indian monsoon. As such, the Indian monsoon could be considered to be weakening and westerlies to be strengthening [1]. Thus, data from the Shiquanhe station could not be used for water balance calculation. Thirdly, the quality of evaporation data in the three stations obtained using the evaporating dish is poor (data for many months were missing since 2002), and therefore, these data could not be used for water balance calculation during 2001–2013. Given all these circumstances, we could not determine the contribution of each factor during different periods, and the dominating factor responsible for lake area expansion. However, this study shows that decreased APE in lakes, increased AP, and increased meltwater from glaciers and frozen soil due to global warming all contributed to lake area expansion.

## Conclusions

In this work, glacial and lake changes in the Songmuxi Co Basin of the Southern Karakoram Mountains were detected based on 1: 50000 topographic maps, Landsat MSS/TM/ETM+/OLI remote sensing data and GIS techniques. The MAT, AP, APE at the Shiquanhe, Hetian and Yutian stations from 1968 to 2013 were used to analyze climate change and its impact on glacier and lake area changes. The results showed that the glacial area loss of the Songmuxi Co basin, Zepu Co basin, Mang Co basin and Unnamed Co was -1.98±0.02 km^2^, -5.39±0.02 km^2^, -0.01±0.02 km^2^ and -0.12±0.02 km^2^ from during the study period, corresponding to a loss of -1.42%, -2.86%, -1.54% and -1.57%, respectively. The lake area of the Songmuxi Co, Zepu Co, Mang Co and Unnamed Co increases of 7.57±0.02 km^2^, 8.53±0.02 km^2^, 1.35±0.02 km^2^ and 0.53±0.02 km^2^, corresponding to growth of 30.22%, 7.55%, 11.39% and 8.05%, respectively. Rising temperature is the main reason for glacier retreat. Due to the lack of continuous long-term monitoring data, this study presented preliminary results that the decreased APE in lakes, increased AP, and increased meltwater from glaciers and frozen soil due to climate warming all contributed to lake area expansion.

## Supporting Information

S1 Dataset Dataset of lake area. (TIF)
(RAR)
